# Recovery of ovarian function by human embryonic stem cell-derived mesenchymal stem cells in cisplatin-induced premature ovarian failure in mice

**DOI:** 10.1186/s13287-020-01769-6

**Published:** 2020-06-26

**Authors:** Sook Young Yoon, Jung Ah Yoon, Mira Park, Eun-Young Shin, Sookyung Jung, Jeoung Eun Lee, Jin Hee Eum, Haengseok Song, Dong Ryul Lee, Woo Sik Lee, Sang Woo Lyu

**Affiliations:** 1 Fertility Center of CHA Gangnam Medical Center, CHA University, 569 Nonhyun-ro, Gangnam-Gu, Seoul, 06125 South Korea; 2 CHA Advanced Research Institute, Seongnam-si, South Korea; 3 Department of Biomedical Science, CHA University, Seongnam-si, South Korea

**Keywords:** Human embryonic stem cell-derived MSC, Chemotherapy-induced premature ovarian failure, Recovery of ovarian function, Ovarian stromal cell apoptosis

## Abstract

**Background:**

Clinical use of mesenchymal stem cells (MSCs) requires a uniform cell population, and their harvesting is invasive and produces a limited number of cells. Human embryonic stem cell-derived MSCs (hESC-MSCs) can differentiate into three germ layers and possess immunosuppressive effects in vitro. Anticancer treatment is a well-known risk factor for premature ovarian failure (POF). In this study, we investigated the effect of hESC-MSC on recovery of ovarian function in cisplatin-induced POF in mice.

**Methods:**

Female mice received intraperitoneal cisplatin for 10 days. On day 12, CHA15-derived hESC-MSCs were transplanted into the mice by tail vein injection. An injection of PBS served as the negative control. Ovaries were removed 28 days after transplantation for assessment of ovarian histology, immunostaining, and fertility testing by superovulation and in vitro fertilization. hESC-MSC transplantation into mice with cisplatin-induced damage restored body weight and ovary size.

**Results:**

Mean primary and primordial follicle counts in the hESC-MSC group were significantly improved compared to the PBS group (*P* < 0.05), and counts of zona pellucida remnants, an apoptotic sign in ovarian follicles, were significantly reduced (*P* < 0.05). TUNEL assays and cleaved PARP immunostaining indicated apoptosis, which led to loss of ovarian stromal cells in negative control mice, while Ki-67 was higher in the hESC-MSC group and in non-cisplatin-treated controls than in the PBS group. Ovulation was reduced in the PBS group but recovered significantly in the hESC-MSC group. Rates of blastocyst formation from ovulated eggs and live births per mouse also recovered significantly in the hESC-MSC group.

**Conclusions:**

hESC-MSC restored structure and function in the cisplatin-damaged ovary. Our study provides new insights into the great clinical potential of human hESC-MSC in treating POF.

## Background

Premature ovarian failure (POF) after chemotherapy is a major long-term adverse effect of anticancer treatment. POF increases the risk of infertility and degenerative health problems, including cardiovascular disease, osteoporosis, and cognitive impairment [1–4]. The impact of chemotherapy on ovarian function varies with patient age at the time of treatment, anticancer drug type, and dose. Chemotherapy may be especially problematic for young women, because loss of ovarian reserve is strongly related to the risk of infertility. Therefore, the preservation of fertility and ovarian function should be a major consideration for chemotherapy in women of reproductive age.

Depending on the woman’s condition, various ways to preserve fertility may be available before or during chemotherapy, including cryopreservation of ovarian tissue, oocytes, or embryos, as well as ovarian protection with gonadotropin-releasing hormone agonists (GnRHa) [5]. However, more studies are required to evaluate feasibility, safety, and efficacy of fertility-preserving methods, and improved therapeutic strategies to prevent chemotherapy-induced gonadal toxicity are needed for these patients.

As a potential alternative therapeutic modality, stem cells have recently been applied to repair and restore normal function of injured tissues or organs. Among such modalities, therapy with mesenchymal stem cells (MSCs), multipotent adult stem cells found in various tissues, is a new option for regenerative therapy that is currently under evaluation in numerous clinical trials worldwide. The exact mechanism by which MSCs mediate the rescue and repair of injured organs and tissues remains largely elusive. Increasing evidence suggests that the therapeutic potential of MSCs could be attributed not only to their engraftment and differentiation but also to other mechanisms such as paracrine release of protein/peptides and hormones, mitochondrial transfer by way of tunneling nanotubes or microvesicles, or the transfer of exosomes or microvesicles containing RNA and other molecules [6].

MSC therapy has also been considered a new option to treat female infertility. To date, many studies in animal models of POF have verified that the administration of MSCs obtained from various cell types protected ovarian function, demonstrating the possibility to restore ovarian function and structure [7]. Among many types of MSCs, human embryonic stem cell-derived MSCs (hESC-MSCs) have many advantages. First, invasive methods are not required to obtain a sufficient quantity of cells. Human embryonic stem cells (hESCs) can be expanded in vitro without limit and without undergoing senescence, so that hESC-MSCs have a more uniform population characteristic and higher growth rate than MSCs generated from other sources [8, 9]. In these regards, hESC-MSCs are especially suitable for use in regenerative therapy.

In this study, we evaluated for the first time the restorative effects of intravenously injected hESC-MSCs on structure and function in mouse ovaries previously injured by cisplatin.

## Methods

### Animal experiments

Seven-week-old ICR or C57BL/DBA F1 hybrid female mice were purchased from Samtako (Seoul, Korea). Animals were housed in the Animal Care Facility of CHA University according to institutional guidelines for laboratory animals under temperature- and light-controlled conditions with a 12-h daily cycle and were fed ad libitum. All animal experiments were approved by the Institutional Animal Care and Use Committee (IACUC 170138).

### Establishment of a murine model of POF and treatment with hESC-MSCs

**Fig. 1:**
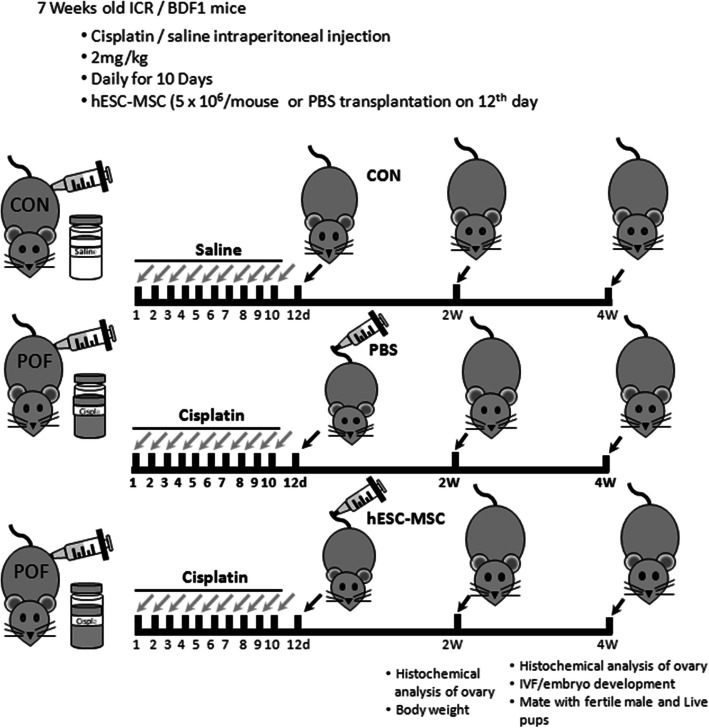
Schematic description of the experimental design. Cisplatin (2 mg/kg) was administered by intraperitoneal injection for 10 days. On day 12, hESC-MSCs (5 × 10^6^/mouse) were transplanted by tail vein injection. Experimental analyses were performed after 2 and 4 weeks

To establish the POF model, mice were injected intraperitoneally with 2 mg/kg cisplatin (Sigma-Aldrich, St. Louis, MO, USA) daily for 10 days, according to Chang et al. [10]. A normal control group (Con) did not receive cisplatin. On the twelfth day of cisplatin administration, mice were randomly divided into treatment groups that received either hESC-MSC (passage 8~10, CHA Stem Cell Institute, Korea) or Dulbecco’s phosphate-buffered saline (PBS, Hyclone, GE Healthcare Life Sciences, Marlborough, MA, USA), by tail vein injection with anesthetization using 2, 2, 2-tribromoethanol (Avertin, Sigma-Aldrich) (Fig. 1). To track transplanted cells in vivo, the cells were pre-labeled overnight with 5 μM CellTracker™ CM-DiI (Molecular probes, Eugene, OR, USA) and 20 μg/ml Molday ION Rhodamine B (MIRB, BioPAL, Inc., Worcester, MA, USA). hESC-MSC were detached from the dish with 0.05% trypsin/EDTA (Gibco-BRL) and centrifuged with PBS three times to remove the medium. hESC-MSCs were diluted 5 × 10^6^/200 μl/mouse with dPBS. After 7 or 14 days, mice were weighed and sacrificed. Both ovaries were removed, fixed, assessed for ovarian histology, and immunostained as described below.

### Differentiation of hESCs into MSCs

All experimental procedures related to the use of hESCs were performed under authorization from the Institutional Review Board for Human Research at the CHA University, Seongnam, Korea, and the National IRB. The culture and differentiation conditions of embryonic stem cell line CHA-hES15 (Korea Stem Cell Registry No. hES12010028) [9] are provided in the Supplementary Methods. Briefly, to form embryoid bodies (EBs), a single hESCs colony was fragmented into two to three small clumps and transferred to a Petri dish (Corning, NY, USA) containing DMEM/F12() supplemented with 20% (v/v) KnockOut serum replacement, 1% (v/v) non-essential amino acids, and 0.1% (v/v) β-mercaptoethanol. In the first differentiation period, EBs were cultured in 1 μM TGF-β inhibitor SB431542 (Sigma-Aldrich) for 15 days. To isolate the mesenchymal progenitor cells (MPCs), mature EBs were transferred after 15 days to 0.1% gelatin (Sigma-Aldrich)-coated 6-well culture dishes, with MSC induction medium, which contained 10% fetal bovine serum (FBS) in DMEM low glucose (Gibco-BRL, Grand Island, NY, USA). After 14 days, outgrown MPCs were detached from the dish by treatment with 0.05% trypsin/EDTA (Gibco-BRL), then transferred to 0.1% gelatin-coated T75 flasks (Thermo Fisher Scientific, Waltham, MA, USA). To induce differentiation into mature MSC by serial sub-passaging, cells were cultured in MSC induction medium for 1 day, then the medium was changed to MSC expansion medium composed of DMEM/F12 containing 10% FBS, 1% non-essential amino acids (Gibco-BRL), and 0.1% β-mercaptoethanol. When MSCs had exhibited homogeneous fibroblast-like morphology for four to five passages, the cells were stored in a 30:60:10 mixture (v/v) of MSC expansion medium/FBS/dimethyl sulfoxide under liquid nitrogen.

**Fig. 2:**
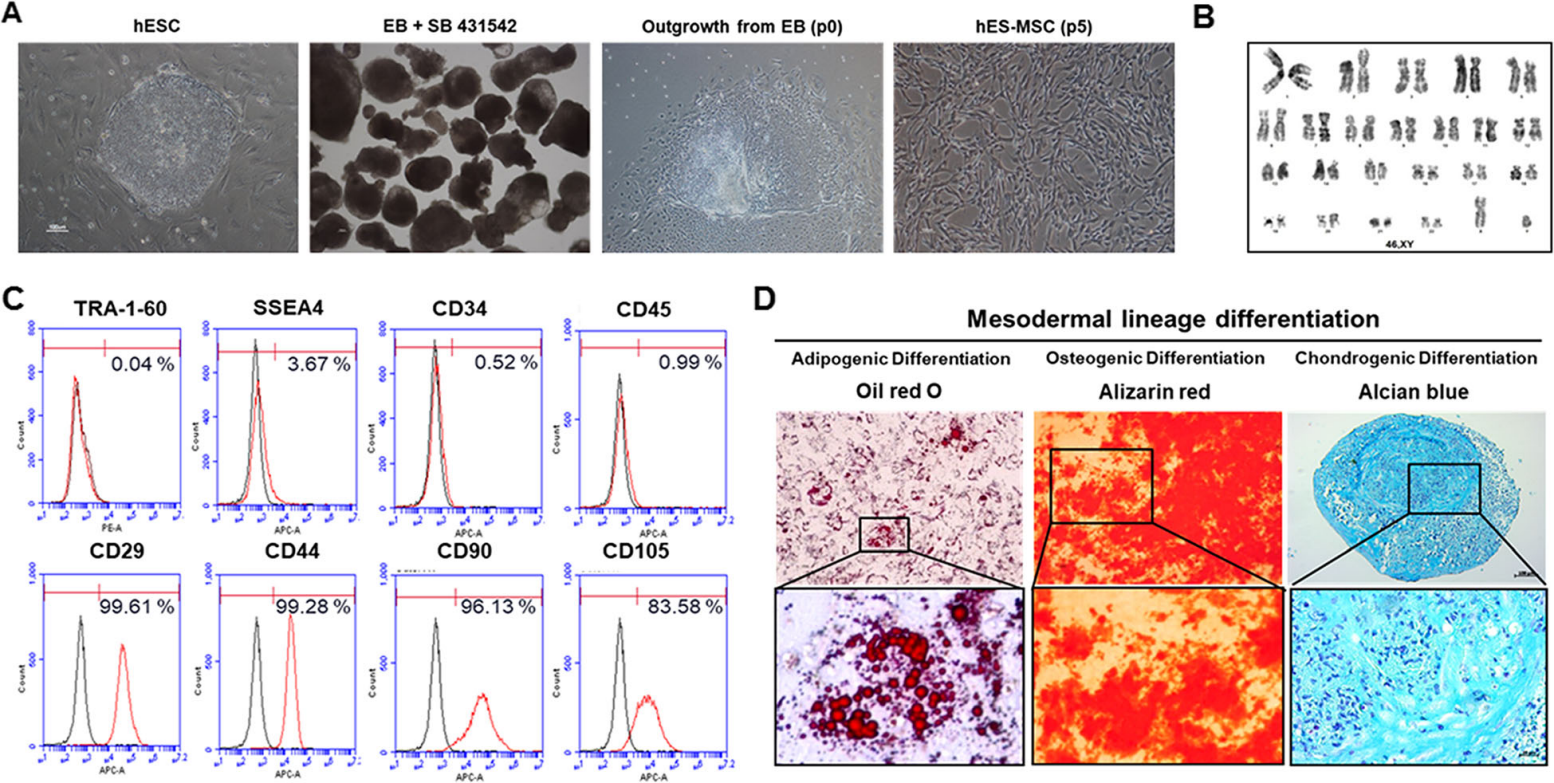
Generation and characterization of mesenchymal stem cells (MSC) from human embryonic stem cells (hESCs). **a** The typical morphology of hESC-MSC. Scale bar = 100 μm. **b** Karyotype of MSCs. **c** Surface antigen profiling in hESC-MSC by FACS. TRA-1-60 and SSEA4 are markers of pluripotency, CD34 and CD45 are hematopoietic markers, and CD29, CD44, CD90, and CD105 are MSC markers. **d** hESC-MSCs have the capacity to differentiate into adipocytes, osteocytes, and chondrocytes. Experimental details are described in Supplementary Methods. Magnified images of each differentiation are shown at the bottom. Scale bar = 100 μm

The characteristics of hESC-MSCs are provided in Fig. 2. Cytogenetic analysis of hESC-MSCs, flow cytometry analysis of MSC cell surface markers, and demonstration of the cells’ capacity to differentiate into adipocytes, osteocytes, and chondrocytes are described in Supplementary Methods.

### Histologic staining and follicle counting

Ovaries were fixed with 4% formaldehyde for 24 h, embedded in paraffin, serially sectioned (5-μm thickness), and stained with hematoxylin and eosin (H&E) to evaluate follicle growth. Follicle counts were conducted on serially cut sections, counting every tenth section, in five to eight mice per group. The mean count per section was calculated. Follicles were counted at different stages using an epifluorescence microscope (Axio Imager 2, Carl Zeiss) with the ZEN image program. Follicle stages were classified according to a previous study [10], as follows: A primordial follicle was defined as an oocyte surrounded by a single layer of flattened, squamous pre-granulosa cells; a primary follicle was an oocyte surrounded by a single layer of cuboidal granulosa cells; a secondary follicle was defined as having two or more layers of cuboidal granulosa cells with no visible antrum; antral follicle was defined as having an antral space with follicular fluid; and the corpus luteum was composed of *lutein cells.* Zona pellucida remnants (ZPRs) were counted to represent growing follicles that had developed a ZP but had consequently undergone atresia [11].

### Detection of apoptosis and proliferation of ovarian tissue

To detect apoptosis of ovarian tissue, deparaffinized tissue sections were permeabilized with 10 μg/ml proteinase K in 10 mM Tris HCl and analyzed by the terminal deoxynucleotidyl transferase-mediated dUTP nick end labeling (TUNEL) assay according to the manufacturer’s instructions (Roche Diagnostics Ltd., Indianapolis, IN, USA). The samples were counterstained with 4′, 6-diamidino-2-phenylindole (DAPI, Molecular Probes). Ovarian stromal cells with TUNEL-positive follicles were observed. To investigate the proliferation of ovarian tissues, deparaffinized tissue sections were blocked with a protein blocking solution (Dako North America, Carpinteria, CA, USA) for 1 h at room temperature. Sections were incubated overnight with Ki-67 antibody (Abcam, Cambridge, MA, USA) at 4 °C overnight. The secondary antibodies were Alexa 555-conjugated goat anti-rabbit and DAPI staining for nucleus. Sections were observed and captured images with an epifluorescence microscope (Axio Imager 2, Carl Zeiss) using the image program ZEN.

### Tracking of transplanted hESC-MSCs

To detect Molday ION Rhodamine B, deparaffinized tissue sections were examined with Prussian blue staining with potassium ferrocyanide (Sigma-Aldrich) and observed on light microscopy (Axio Imager 2).

### DNA extraction from ovary and nested PCR for human *SRY* gene

Genomic DNA was extracted from mouse tissues, including liver, skeletal muscle, ovary, uterus, and spleen, with LaboPass™ Tissue Mini kit (Cosmo Genetech Co., Ltd., Seoul, Korea), according to the manufacturer’s protocols. Nested PCR reactions (20 μl) contained 1 μl each primer (5 pM), 2 μl 10× Taq reaction buffer (with 25 mM MgCl_2_), 0.4 μl of 10 mM dNTP mix, and 0.1 μl of SolG™ Taq DNA polymerase (5 U/μl, SolGent Co., Ltd.). For the first round of amplification, reactions contained 100 ng genomic DNA template and the primers hSRY-1st F (GTAAAGGCAACGTCCAGGATAGAG) and hSRY-1st R (GCATCTAGGTAGGTCTTTG -TAGCC). For the second round of amplification, reactions contained 1 μl first-round PCR product and the primers hSRY-2nd F (GCGACCCATGAACGCATT and hSRY-2nd R (AGTTTCGCATTCTGGGATTCTCT). Mouse gapdh (mgapdh, F, TCCCCTTAGTTCGAGGGACT, and R, ACATCACCCCCATCACTCAT) was used for control gene. Thermal cycling was performed with a SimpliAmp Thermal Cycler (Applied Biosystems, Thermo Fisher Scientific). The cycling conditions comprised an initial denaturation step at 95 °C for 3 min, 30 cycles of 30-s denaturation at 95 °C, 30-s annealing at 60 °C, and 30-s extension at 72 °C, then a final extension at 72 °C for 5 min. For the second round of amplification, initial denaturation was at 95 °C for 2 min, followed by 30 cycles of amplification and a final extension as in the first round. One positive control (genomic DNA extracted from donated human blood with written consent, under approval by the institutional review board (IRB) of CHA University (1044308-201803-BR-014-02)) and one negative control (genomic DNA extracted from mouse tissue) were included in each PCR analysis. The amplicons were mixed with Loading Star dye, and analyzed by 1.5% agarose gel electrophoresis beside a 100-bp DNA ladder (both from Dyne Bio, Seongnam-si, Korea).

### Western blotting

Ovaries were homogenized in lysis buffer (PRO-PREP™ Protein Extraction Solution, Intron, Korea), centrifuged at 14,000×*g* for 15 min; then, supernatants were diluted to 1 μg/μl with 4× sample buffer (Bio-Rad, Hercules, CA, USA) and frozen at 20 °C. Proteins samples were boiled for 3 min. The extracted proteins were separated by 12% sodium dodecyl sulfate polyacrylamide gel electrophoresis and transferred to PVDF membranes. Membranes were blocked with 5% non-fat dry milk in PBS containing 0.1% Tween 20, then incubated overnight with cleaved PARP Asp214 antibody (Cell Signaling, Danvers, MA, USA) at 4 °C. Horseradish peroxidase-conjugated secondary antibodies were incubated for 1 h at room temperature, and immunoreactivity was detected using enhanced chemiluminescence reagent (Santa Cruz Biotechnology, Santa Cruz, CA, USA) and recorded on Amersham Hyperfilm ECL (GE Healthcare, Buckinghamshire, UK). Visualized bands were quantified by densitometry with NIH Image J software (https://imagej.nih.gov/ij/docs/faqs.html). Intensities of bands were expressed relative to that of the control.

### In vitro fertilization and embryonic development

Four weeks after hESC-MSC transplantation, BDF1 mice were induced to superovulate with 10 IU pregnant mare’s serum gonadotropin (PMSG, Sigma-Aldrich) and human chorionic gonadotropin (hCG, Sigma-Aldrich). Cumulus-enclosed mature eggs were collected from the oviduct 13 h later. Cumulus cells were removed with 0.1% hyaluronidase (Sigma-Aldrich). Eggs were fertilized in human tubal fluid medium with epididymal sperm obtained from 12-week-old fertile male mice. The pronuclear embryos were collected 6 h after in vitro fertilization. Fertilized embryos were cultured in KOSM (EMD Millipore) for 5 days to the blastocyst stage. Hatching rates were examined on days 6 and 7. Differential staining was performed to examine inner cell mass (ICM) and trophectoderm (TE) cells, using anti-Oct3/4 (Santa Cruz Biotechnology) and DAPI.

To obtain live pups, the mice were fertilized 4 weeks after transplantation by introducing fertile males for 10 days.

### Statistical analyses

All experiments were repeated at least three times. Quantitative variables are represented as mean ± standard error of the mean (SEM). All statistical analyses were performed using either ANOVA with Tukey’s post hoc test or the Student’s *t* test, using GraphPad Prism 7 (GraphPad Software, Inc., La Jolla, CA, USA). A comparison of the survival curves analyzed by chi-square test using GraphPad Prism 7. *P* values < 0.05 or < 0.0001 were considered statistically significant.

## Results

### Characterization of hESC-MSCs

It has been shown that hESC-MSCs are able to differentiate into adipocytes, chondrocytes, and osteocytes [9]. Herein, hESC-MSCs showed homogeneous fibroblast-like morphology in the fourth and/or fifth passage, with clear cell surface expression of CD29, CD44, CD90, and CD105 at frequencies of greater than 80–90% (Fig. 2a, c), as well as 22+XY chromosomal status (Fig. 2b). In contrast, hESC-MSCs minimally expressed hematopoietic cell markers (CD34 and CD45, < 1%) and hESC markers (Tra-1-60 and SSEA4, < 4%) (Fig. 2c).

### Establishment of a murine model of cisplatin-induced POF and hESC-MSC transplantation

**Fig. 3:**
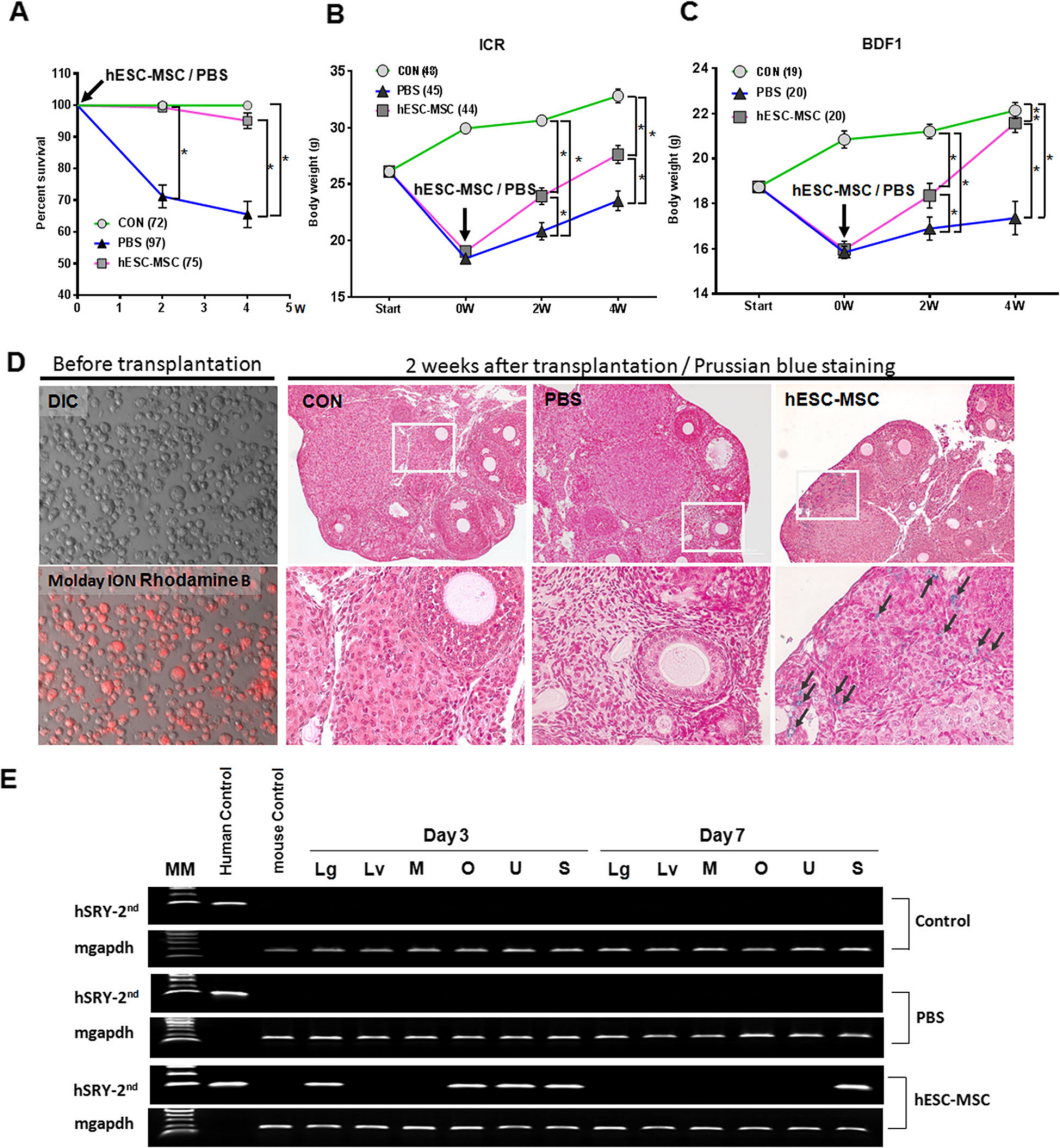
Improved survival rate and changes in body weight of mice treated with cisplatin by hESC-MSC transplantation and tracking of hESC-MSC distribution in vivo. Total numbers of mice were 72, 97, and 75 in the control (CON), PBS, and hESC-MSC treatment groups, respectively. **b**, **c** Body weights were measured each day during cisplatin administration and again 2 and 4 weeks after hESC-MSCs transplantation. *P < 0.05, **P < 0.001. **d** Tracking of hESC-MSC distribution in vivo by Molday ION B with Prussian blue staining. **e** Nested PCR detection of human *Sry* gene. The control gene was mouse GAPDH (mgapdh). MM, molecular marker; L, liver; M, muscle; O, ovary; U, uterus; S, spleen

Animals were weighed before treatment with either cisplatin at 2.0 mg/kg or saline (control). Overall survival rate of treated mice was 65.5% (63 out of 97 mice). Significant weight loss began on day 5 and mortality occurred on day 10 in mice that received cisplatin (Fig. 3a–c). Following cisplatin administration, mice were randomly divided into two experimental groups that were administered either PBS or hESC-MSCs via the tail vein. Mouse survival at 2 weeks (Fig. 3a) in the PBS group was significantly (*P* < 0.0001) lower (73 out of 97 mice, 71.1 ± 3.6%) than that in the hESC-MSC group (74 out of 75 mice, 99.3 ± 0.6%). Final survival rate at 4 weeks in the PBS and the hESC-MSC group was 65.5 ± 4.1% (63 out of 97 mice) and 96.9 ± 2.0 (73 out of 75 mice). Most of the deaths occurred within 5 h after the transplant procedure that was conducted with anesthesia. Following the transplant procedure, mice that received hESC-MSCs recovered body weight significantly more than PBS controls in both ICR and BDF1 strains (Fig. 3b, c).

### Tracking of hESC-MSCs in vivo

To track hESC-MSCs in vivo after transplantation, deparaffinized ovary sections were investigated using a fluorescence microscope. At 2 to 4 weeks after transplantation, no fluorescence signal was detected by CellTracker CM-DiI and Molday ION Rhodamine B. However, 2 weeks after transplantation, hESC-MSCs stained with Prussian blue were observed in non-follicular stromal cells of ovaries only in hESC-MSC-transplanted mice and not in the control or PBS groups (Fig. 3d). PCR analysis for the human gene *Sry*, which is indicative of the distribution of hESC-MSCs, demonstrated the presence of *Sry* in the hESC-MSC group but not in the control or PBS groups (Fig. 3e). *Sry* was detected in ovary, uterus, and spleen tissues on day 1 after transplantation and only in the spleen on day 7.

### Restoration of ovarian structure and function

**Fig. 4:**
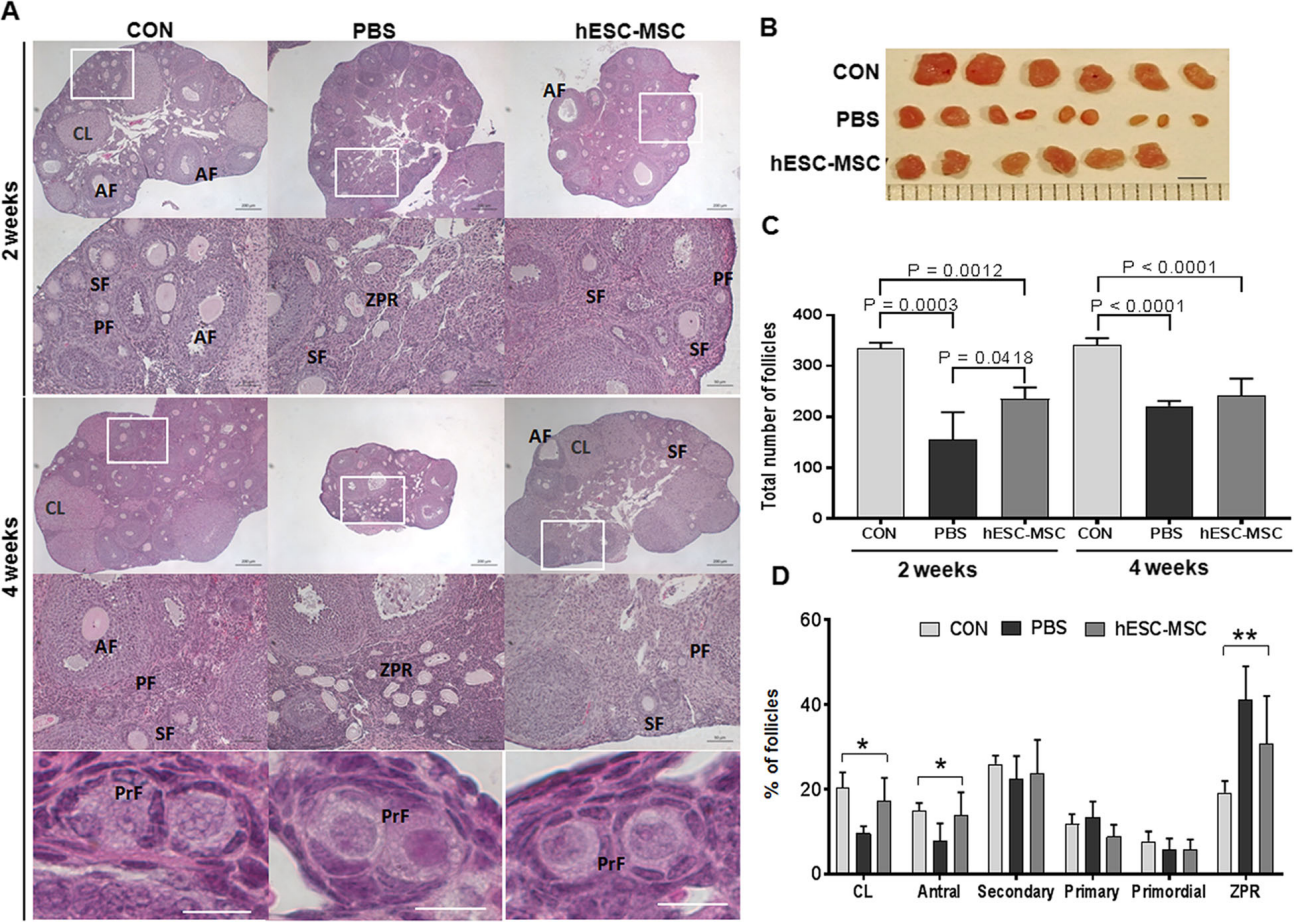
Recovery of ovarian structure by hESC-MSC transplantation in cisplatin-induced ovarian failure. **a** Ovarian histology was analyzed 2 and 4 weeks after transplantation using H&E staining. Scale bar = 200 or 50 μm. PrFs in insets were captured from other section. **b** Ovaries were removed from mice in the control, cisplatin + PBS, and cisplatin + hESC-MSC transplantation groups. Scale bar = 2 mm. **c** Total number of follicles per ovary. **d** Percentages of each follicle type per ovary. *P < 0.05, **P < 0.001; CL, corpus luteum; AF, antral follicle; SF, secondary follicle; PF, primary follicle; PrF, primordial follicle; and ZPR, zona pellucida remnant

To further examine the effects of hESC-MSC transplantation on ovarian function, ovaries were analyzed histologically. In every experiment, treatment with hESC-MSCs improved ovarian size compared to the PBS group. Two weeks after transplantation, PBS and hESC-MSC groups showed significantly reduced total follicle counts compared to control, with the PBS group showing the lowest number. However, at 4 weeks, total follicle numbers in the PBS group had recovered as much as in the hESC-MSC group (Fig. 4b, c). The numbers of corpus luteum and antral follicles recovered to control levels in the hESC-MSC group but not in the PBS group (Fig. 4d, *P* < 0.005). In the PBS group, more than 40% of follicles were characterized as zona pellucida remnants, which are known to be apoptotic follicles of atresia (Fig. 4d, *P* < 0.0001).

**Fig. 5:**
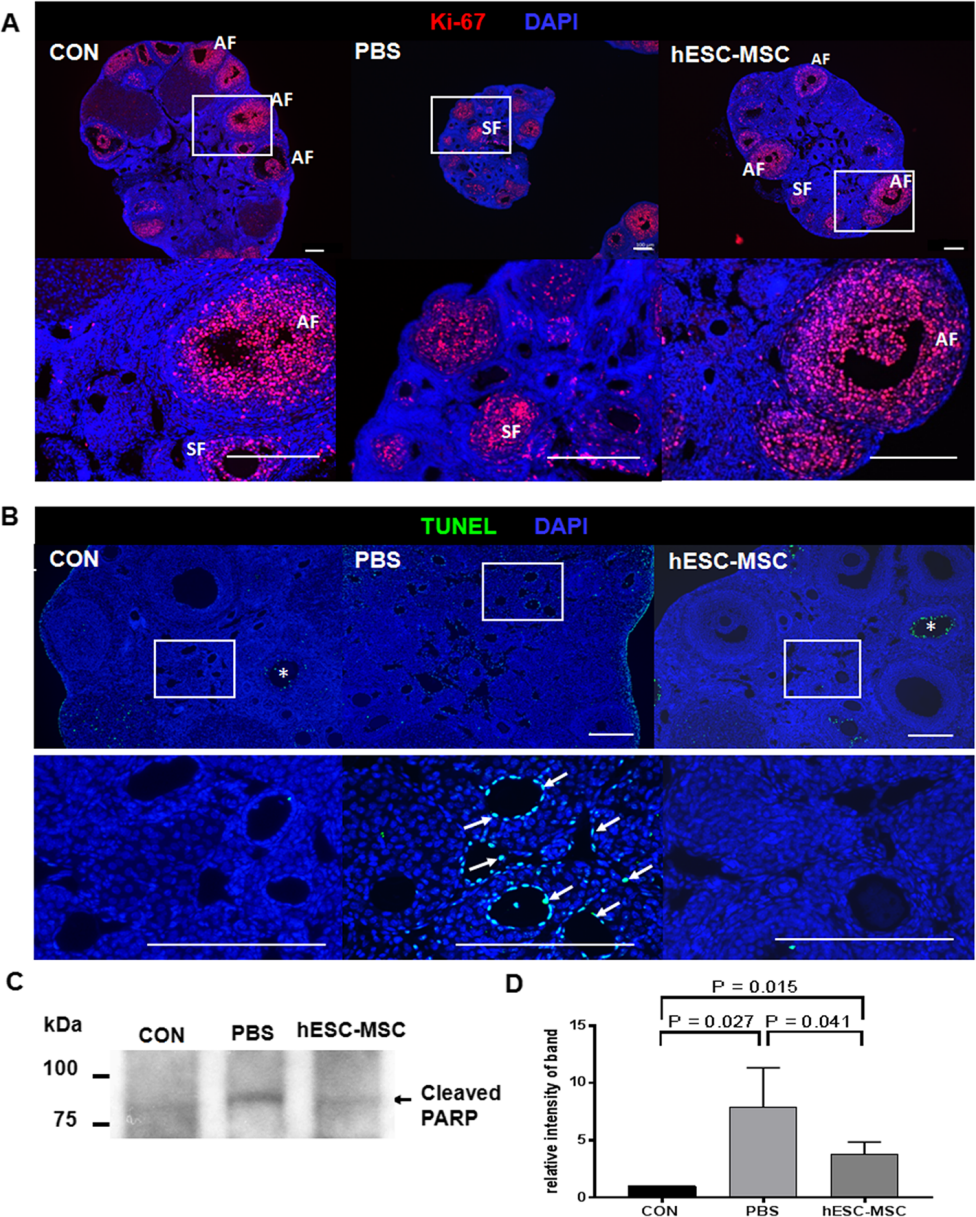
Effect of hESC-MSCs on ovarian stromal and granulosa cells injured by cisplatin. **a** Immunofluorescence of Ki-67 (red) represents granulosa cell proliferation in the ovary. **b** TUNEL detection (green) demonstrates apoptotic signals in ovarian stromal cells. **a**, **b** Blue represents DAPI-stained nuclei. Scale bar = 100 μm. **c**, **d** Western blot results with relative band intensities calculated from three different blots

Proliferation of granulosa cells in secondary and antral follicles was observed by Ki-67 immunostaining in all experimental groups (Fig. 5a). However, TUNEL assays showed significant apoptosis occurring in ovarian stromal cells of the PBS group, but not in the control or hESC-MSC groups (Fig. 5b). Likewise, cleaved PARP detected by western blotting was significantly higher in the PBS group compared to control and hESC-MSC groups (*P* = 0.0027 and 0.041, respectively, Fig. 5c, d). However, the western blot assay indicated that apoptotic activity in hESC-MSC-treated mice did not recover completely to control levels (*P* = 0.015).

### Rescue of ovulation of mature eggs, embryonic development, and live birth by hESC-MSC

**Fig. 6:**
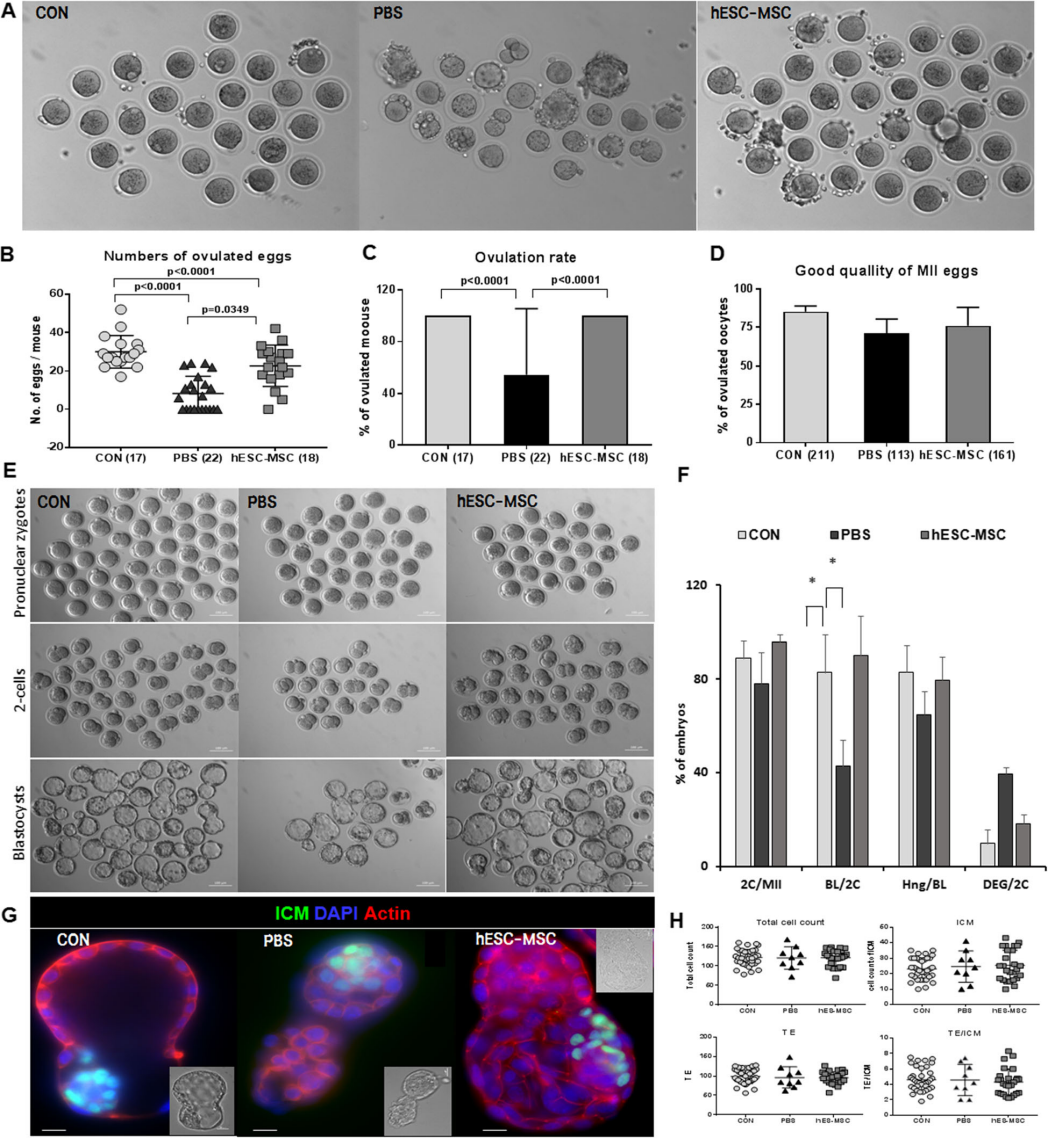
Rescue of ovarian function by hESC-MSC transplantation after cisplatin injury. **a** Ovulated eggs from control, PBS, and hESC-MSC-transplanted mice at 4 weeks. **b** The average number of eggs ovulated per mouse. **c** Ovulation rate, calculated as the fraction of the number of mice in the group that ovulated. **d** Quality of ovulated eggs. **e** Embryonic development to blastocyst from ovulated eggs of treated mice following in vitro fertilization. **f** Percentage of fertilization with pronuclear zygotes, two cells, and blastocyst formation and hatching after in vitro fertilization. **g** Hatching blastocysts were immunostained for Oct3/4 (green) of the ICM, actin-phalloidin (red) in TE, and nuclear staining with DAPI (blue). **h** Total cell numbers in ICM and the TE and the ratio of TE cells to ICM cells. Scale bar = 100 μm (**e**) and 10 μm (**g**)

All control mice ovulated in response to superovulation, with an average of 29.9 ± 2.0 eggs (Fig. 6a, b, mean ± SEM), whereas only 12 of 22 mice (54.5%) ovulated in the PBS group (Fig. 6c), with an average of just 8.2 ± 1.9 eggs (Fig. 6b). In contrast, all hESC-MSC-treated mice ovulated, an average of 22.7 ± 2.5 eggs (Fig. 6a–c, *P* < 0.0001 vs. control, *P* = 0.0034 vs. PBS). There were no significant differences in the quality of ovulated eggs among the treatment groups (Fig. 6d). The rates of fertilization of the ovulated eggs and blastocyst formation from two cells were also rescued by hESC-MSC transplantation (Fig 6e, f). In blastocysts, ICM cell counts and the TE did not differ among experimental groups (Fig. 6g, h).

**Fig. 7:**
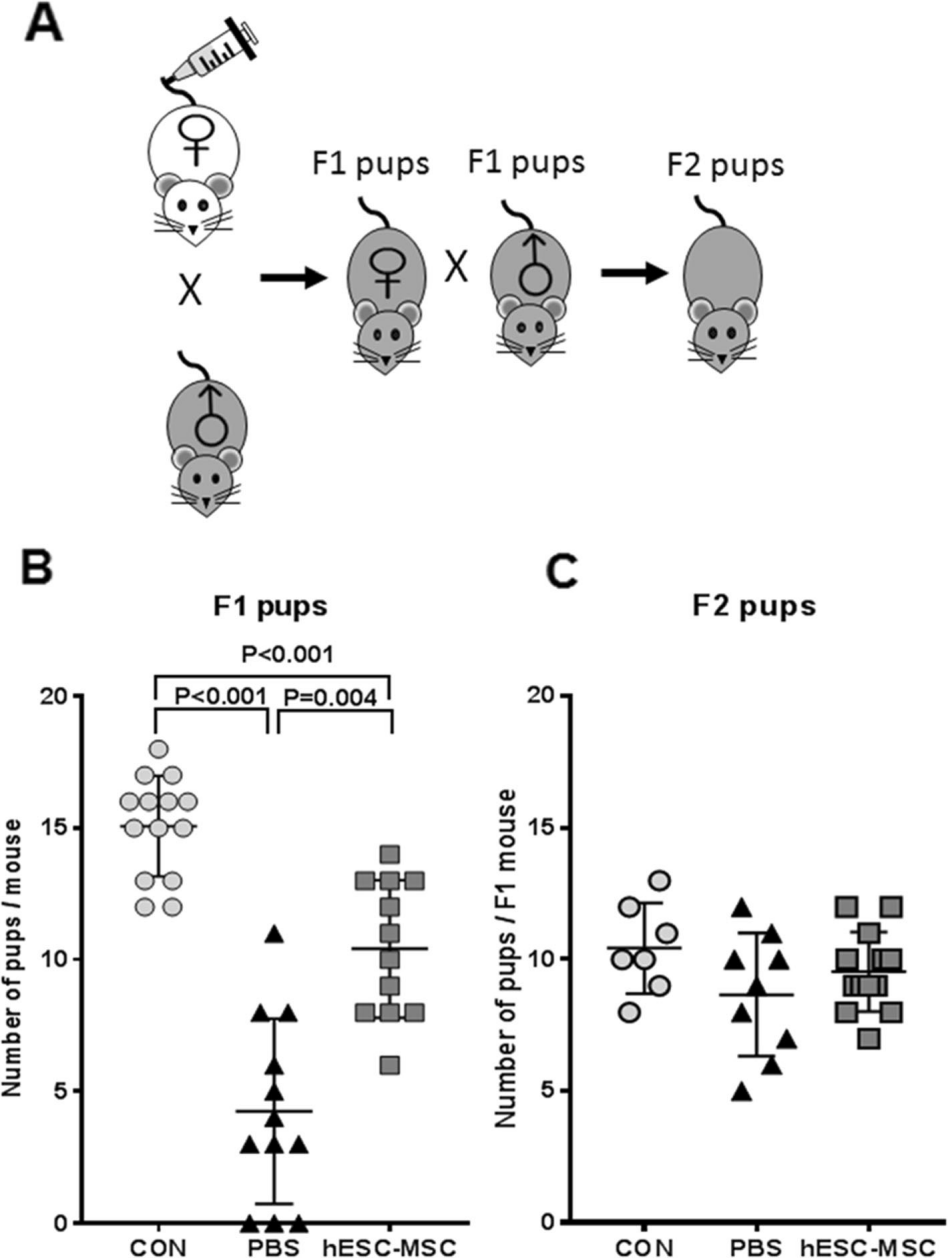
Fertility restoration by hESC-MSC transplantation in mice with cisplatin-induced ovarian failure. **a** Reproductive outcomes (offspring obtained) in three mating experiments with fertile males. Mating occurred 4 weeks after hESC-MSC transplantation. Mean litter size per pregnant mouse for generation F1 (**b**) or F2 (**c**). Data represent mean ± SEM

Four weeks after hESC-MSC transplantation, female mice were mated with fertile BDF1 male mice. One mouse in the PBS group did not become pregnant for 3 months. Live births per mouse were 15.0 ± 0.5, 5.1 ± 1.0, and 10.1 ± 1.2 for the control, PBS, and hESC-MSC groups, respectively. The live birth rate was significantly lower for the PBS group compared to either the control or the hESC-MSC group (Fig. 7a, b). Two mice in the PBS group delivered one and two pups each that died on the day of birth. However, female mice treated with hESC-MSC delivered 6–13 pups each and cared for the offspring normally.

Mating of F1 mice produced an F2 generation. Numbers of F2 pups born alive did not differ among the three experimental groups (Fig. 7c).

## Discussion

POF is a possible complication of chemotherapy that significantly impacts the quality of life of premenopausal women. Moreover, loss of ovarian reserve is strongly related to the risk of female infertility, and consequently, fertility issues may be a major concern to women of reproductive age who require chemotherapy. Although several options, including ovarian tissue, oocyte, and embryo cryopreservation, have been applied, these patients need improved therapeutic strategies to prevent chemotherapy-induced ovarian failure. In the present study, we evaluated for the first time the restorative effects of hESC-MSCs on structure and function in cisplatin-injured ovaries of mice. We showed that hESC-MSC administration following chemotherapy may restore ovarian structure and function and enhance pregnancy capacity, suggesting that hESC-MSC may prove effective for ovarian protection and fertility preservation during chemotherapy in female cancer patients.

Cisplatin, one of the most common anticancer drugs, is used to treat various pediatric and adult cancers. However, cisplatin is related with many side effects, such as emesis, nephrotoxicity, neurotoxicity, myelosuppression, immunosuppression, and ototoxicity [12]. In the present study, the higher mortality was represented in the PBS group with kidney lesions compared to the control and hESC-MSC group (Fig. 3a, [10]). However, cisplatin kills cancer cells by inducing the formation of inter-and intra-strand DNA adducts. Though known to cause intermediate gonadal toxicity, cisplatin-based chemotherapy can lead to POF and irreversible infertility in women of reproductive age [13, 14]. Although the mechanism of ovarian failure after cisplatin administration has not been established, studies in mice and rats showed that high doses of cisplatin induced overactivation of dormant primordial follicles, resulting in loss of ovarian reserve. Thus, for women of reproductive age who receive chemotherapy with agents such as cisplatin, it is very important to develop adjuvant therapies that can reduce POF.

The impacts of chemotherapy on ovarian function vary with age at the time of treatment, type of treatment, and dose of anticancer drug. Various approaches to preserve fertility in women with cancer may be called for, depending on patient age, current success rate, the requirement to delay cancer treatment, required ovarian stimulation, sperm requirement, and the risk of reintroducing malignant cells. These approaches may include cryopreservation of embryos, oocytes, immature oocytes, or ovarian tissue, transplantation, and ovarian protection with GnRHa [5]. However, more studies are required to evaluate feasibility, safety, and efficacy of these fertility-preserving methods and identify better therapeutic strategies to protect against the loss of ovarian reserve in chemotherapy.

Recently, MSC therapy has been considered a new option to treat infertility. To date, many studies in animal models of POF have demonstrated ovarian protection by the administration of MSCs obtained from various cell types. Also, MSCs display a powerful ability to regulate immune responses, including suppression of T cell and B cell proliferation and modulation of natural killer cells and macrophage [15]. For this reason, even in this study, MSCs derived from human embryonic stem cells could be transplanted into mice.

According to a recent review, MSCs derived from bone marrow (BM), adipose tissue (AD), umbilical cord (UC), menstrual blood (Men), skin, and amniotic fluid have been applied to restore fertility in chemotherapeutic agent-induced POF using animal models such as mice, rats, and rabbits [7]. Transplantation of these MSC has shown potential for the restoration of ovarian function and folliculogenesis, diminishment of granulosa cell apoptosis, and recovery of ovarian structure [7]. However, the different assessment methods used in the animal studies make it difficult to compare results obtained with the various types of MSCs, and the exact mechanisms by which they protect against chemotherapy-induced ovarian damage have not been elucidated.

For many types of MSCs, invasive procedures are required to harvest the source cells, and it is difficult to obtain a sufficient quantity of cells with sufficient uniformity for clinical use. Certain types of hMSCs, such as AD-MSC or BM-MSC, have limited proliferative capacity due to replicative senescence [16–18] and tend to be more pro-inflammatory when derived from older donors [19–21]. In addition, higher passage MSCs are more likely to trigger an innate immune response, termed instant blood-mediated inflammatory reaction, in vitro and in vivo, that compromises the survival and function of systemically infused cells [22]. MSCs may be derived instead from hESCs, a limitlessly expandable source that does not senesce in vitro. The ability to consistently generate MSCs from a single hESC source can ensure stem cell uniformity and minimize batch-to-batch variation. The growth rate of hESC-MSCs during early expansion was found to be significantly higher than that of AD-MSC or BM-MSC [8]. In this regard, hESC-MSCs may be useful for regenerative therapy.

It is well known that ovarian follicular development requires both systemic regulation by hormones and intraovarian regulation by various cytokines, growth factors, and intracellular proteins. Ovarian stromal cells and granulosa cells (GCs), in particular, play an important role in follicular growth and maintenance. In the present study of cisplatin-injured mouse ovaries, intravenously delivered hESC-MSCs reduced apoptotic signaling in stromal cells and increased proliferation of granulosa cells. Moreover, hESC-MSC transplantation restored the number of ovarian follicles and reduced the ZPR count, another apoptotic sign in ovarian follicle (Figs. 5 and 6).

Although the mechanism is still unclear, the restorative effects of hESC-MSCs on ovarian stromal cells, GCs, and follicles are believed to be due to their secretion of paracrine factors such as growth factors, cytokines, angiogenic factors, and extracellular matrix proteins [6]. MSCs are known to secrete a variety of factors including epidermal growth factor, vascular endothelial growth factor, insulin-like growth factor I, hepatocyte growth factor, insulin-like growth factor-binding proteins, and transforming growth factor β [23]. These secreted paracrine factors have been demonstrated to restore ovarian function and structure [24, 25], and they may account for the restoration of ovarian function observed in our study. These findings suggest that paracrine activity of hESC-MSCs may influence the ovarian microenvironment, protecting against ovarian toxicity of chemotherapy. Our study demonstrated that hESC-MSC transplantation decreased apoptotic activity in ovarian stromal cells, restoring ovarian structure. It may also restore ovarian function. We observed the rescue of folliculogenesis, including ovulation, fertilization, and embryonic development, with the birth of live pups.

In this study, we performed this experiment to see if implanted MSCs directly affect the ovarian structure and function recovery. The transplanted MSC is inferred to return to various organ tissues (lung, liver, spleen, uterus, ovary, Fig. 3e) when predicting damage to multiple organs in mice due to various side effects of cisplatin. Thus, it appears that very small amounts of transplanted cells have been introduced into the ovaries. On day 3, the SRY gene detected in ovarian tissue was not detected on day 7, which is consistent with studies showing a sharp decrease in fluorescence expression experiments on day 7 [26]. Similarly, only a very small amount of Prussian blue traces for Molday Iron were stained throughout the whole ovarian tissue, which appears to be some cells remaining after transplantation. Therefore, the recovery of ovarian structure and function by MSC in this study can be regarded as the effect of paracrine rather than the direct effect of MSC entering the ovary. Meanwhile, the effect of hESC-MSC transplantation in cisplatin-treated mice was observed immediately after transplantation. Compared to the PBS group, rapid movement and atrophy of the body due to anticancer drugs have been restored, which is thought to be the result of the transplanted cells reaching each organ damaged by the MSC homing effect [26].

POF may occur as a side effect of chemotherapy in premenopausal women. Even if regular menses resume after chemotherapy, patients remain at risk of early menopause and infertility due to cytotoxic damage to the ovarian reserve. In the present study of cisplatin-induced damage, hESC-MSC transplantation protected mice against loss of body weight, as well as ovarian size, and significantly increased the number of eggs ovulated in superovulation, as well as blastocyst formation and the number of live births per mouse. These progeny exhibited a normal rate of production of second-generation pups (F2). Our findings demonstrated that hESC-MSC administration effectively preserved ovarian function and fertility following chemotherapy.

Some issues remain to be resolved. First, hESC-MSCs were found to be equivalent to BM- or AD-MSCs as a source of MSCs for ovarian function [8]. However, ethical concerns regarding the use of human embryo-derived stem cells persist. These concerns may be avoided by the use of induced pluripotent stem cells (iPSCs), an alternative source of MSCs. iPSCs can be obtained with minimally invasive procedures, and recently, autologous or HLA-matched iPSC-derived MSCs were reported to minimize immunological problems [27, 28]. Also, mesenchymal progenitor cells from somatic cell nuclear transfer-derived pluripotent stem cells may benefit endometrial function in the mouse uterus [9]. Safety is a second issue hindering treatment with hESC-MSCs, due to concerns about unwanted, unexpected, or uncontrolled differentiation of hESC-MSCs after transplantation [29]. Options to address this issue that have been introduced in numerous disease models include the use of bio-degradable [30] or biomimetic [31] scaffold-encapsulated MSCs, or of membrane-bound biological nanoparticles (exosomes) secreted by MSCs [32–34].

## Conclusion

In conclusion, this report is the first to demonstrate that hESC-MSC administration following chemotherapy may restore ovarian structure and function, thus enhancing the capacity for pregnancy, in mice with cisplatin-injured ovaries. Our study provides new insights into the great clinical potential shown by hESC-MSCs to alleviate chemotherapy-induced POF.

## Supplementary information

**Additional file 1.** Supplementary materials and methods

## Data Availability

All data generated or analyzed during this study are included in this published article.

## Abbreviations

POF: Premature ovarian failure
hESC-MSCs: Human embryonic stem cell-derived MSCs
GnRHa: Gonadotropin-releasing hormone agonists
ZPRs: Zona pellucida remnants
TUNEL assay: Terminal deoxynucleotidyl transferase-mediated dUTP nick end labeling assay
DAPI: 4′, 6-Diamidino-2-phenylindole
PMSG: Pregnant mare’s serum gonadotropin
hCG: Human chorionic gonadotropin
ICM: Inner cell mass
TE: Trophectoderm
BM: Bone marrow
AD: Adipose tissue
UC: Umbilical cord
Men: Menstrual blood
GCs: Granulosa cells
iPSCs: Induced pluripotent stem cells
