# Supplementary material for: Recovery of ovarian function by human embryonic stem cell-derived mesenchymal stem cells in cisplatin-induced premature ovarian failure in mice
Source: Stem Cell Res Ther. 2020 Jun 26;11:255. doi: 10.1186/s13287-020-01769-6 (PMC7318510; doi:10.1186/s13287-020-01769-6)
Supplement: Supplementary file 1 — Additional file 1. Supplementary materials and methods [file 13287_2020_1769_MOESM1_ESM.docx]

**Supplementary materials and methods**

**Differentiation of human embryonic stem cells (hESCs) into mesenchymal stem cells (MSCs)**

Human embryonic stem cell line CHA-hES15 (Korea Stem Cell Registry No. hES12010028) was cultured on a feeder layer of mouse embryonic fibroblasts (MEFs), in ES maintenance medium composed of Dulbecco's modified Eagle medium: nutrient mixture F12 (DMEM/F12) supplemented with 20% (v/v) KnockOut serum replacement, 1% (v/v) non-essential amino acids, 0.1% (v/v) β-mercaptoethanol, and 4 ng/ml human recombinant basic fibroblast growth factor (bFGF, all from Gibco-BRL, Grand Island, NY, USA).

To form embryoid bodies (EBs), a single hESC colony was fragmented into two to three small clumps using a sterile micro-tip (Axygen, Corning, NY, USA) under microscopic observation. These were transferred to a Petri dish (Corning, USA) containing ES maintenance medium without bFGF (EB medium), for suspension culture.

**Chromosome analysis**

For cytogenetic analysis, hESC-derived MSCs were incubated for 30 min in MSC expansion medium containing 0.2 μg/ml colcemid (KaryoMax, Gibco-BRL). Thereafter, they were treated 30 min with a hypotonic solution (0.7% sodium citrate buffer), then fixed with a 3:1 (v/v) mixture of methanol (Merck, KGaA, Germany) and acetic acid (Sigma-Aldrich). Cells were spread onto a glass slide and dried. Metaphase spreads were stained using the G-bands by trypsin using the Giemsa (GTG)-banding technique, and 20 metaphases were analyzed and karyotyped by two cytogenetic experts. The ideogram was produced by the Ikaros karyotyping system (MetaSystems, Altlussheim, Germany).

**Flow cytometry analysis of cell surface MSC markers**

hESC-MSCs were washed with PBS, treated with 0.05% trypsin-EDTA (Gibco-BRL) for 2 min in a 37°C, 5% CO_2_ incubator, and centrifuged at 1,000 rpm for 5 min. Cells were fixed for 1 hour in pre-chilled 4 % paraformaldehyde solution (EL Bio, Seongnam si, Korea) at 4°C, washed with FACS buffer composed of 2 % (v/v) FBS in PBS, and centrifuged three times. The cell pellet was resuspended in 100 μl FACS buffer containing fluorochrome-conjugated antibody, and incubated at room temperature for 30 min in the dark. Phycoerythrin-conjugated mouse anti-human TRA-1-60 and allophycocyanin (APC)-conjugated mouse anti-human/mouse SSEA4 (Cat. FAB/435A, R&D Systems, Minneapolis, MN, USA) were used as stemness markers, APC-conjugated mouse anti human CD34 and APC-conjugated mouse anti-human CD45 as hematopoietic markers, and APC-conjugated mouse anti-human CD29, APC-conjugated mouse anti-human CD44, APC-conjugated mouse anti-human CD90, and APC-conjugated mouse anti-human CD105 as MSC markers (except as otherwise stated, all antibody conjugates were from BD Pharmingen, San Diego, CA, USA). After incubation, cell samples were washed by addition of 1 ml PBS and centrifugation at 1,000 rpm for 5 min, then analyzed using an Accuri C6 Plus flow cytometer equipped with Cell Quest software (BD Biosciences, San Diego, CA, USA).

**Mesodermal lineage differentiation and characterization**

To demonstrate adipogenic differentiation, hESC-MSC were seeded at 2×10^5^ cells/well into 0.1% gelatin-coated 12-well tissue culture dishes in MSC expansion medium. When fully confluent, adipogenic differentiation medium (StemPro^®^ Adipogenesis Differentiation Kit, Gibco-BRL) was introduced for 21 days, with three media changes per week. After complete differentiation, cells were washed twice with PBS, fixed in pre-chilled 4 % paraformaldehyde solution, then stained with Oil Red O solution (IHC World, Woodstock, MD, USA), according to the manufacturer's instructions. Stained lipid droplets in the cells were visualized and photographed with an inverted microscope (TE2000-U, Nikon, Japan). To demonstrate potential for osteogenic differentiation, these cells were cultured with the StemPro^®^ Osteogenesis Differentiation Kit (Gibco-BRL), then intracellular calcium deposits in osteocytes were stained with alizarin red solution. To demonstrate potential for chondrogenic differentiation, cells were cultured in StemPro^®^ Chondrogenesis Differentiation Kit (Gibco-BRL), then fixed and embedded in paraffin. Chondrocytes in the sectioned samples were demonstrated by staining of cell surface alkaline phosphatase with Alcian blue solution (Cat. IW3000, IHC World) according to the manufacturer's instructions. Stained carboxylated mucins on differentiated chondrocytes were visualized and photographed using a light microscope (Primo Star, Zeiss, Germany).
